# Native T1 values identify myocardial changes and stratify disease severity in patients with Duchenne muscular dystrophy

**DOI:** 10.1186/s12968-016-0292-8

**Published:** 2016-10-28

**Authors:** Laura J. Olivieri, Peter Kellman, Robert J. McCarter, Russell R. Cross, Michael S. Hansen, Christopher F. Spurney

**Affiliations:** 1 Division of Cardiology, Children’s National Health System, 111 Michigan Avenue NW, W3-200, Washington, DC 20010 USA; 2 National Heart, Lung and Blood Institute, National Institutes of Health, Bethesda, MD 20892 USA; 3 Children’s National Health System, Clinical and Translational Science Institute, 111 Michigan Ave NW, Washington, DC 20010 USA; 4 Children’s National Health System, Center for Genetic Medicine Research, 111 Michigan Ave NW, Washington, DC 20010 USA

**Keywords:** Duchenne muscular dystrophy, Cardiovascular magnetic resonance, Cardiomyopathy, Pediatrics, T1 mapping

## Abstract

**Background:**

Duchenne muscular dystrophy (DMD) is an X-linked, inherited disorder causing dilated cardiomyopathy with variable onset and progression. Currently we lack objective markers of the effect of therapies targeted towards preventing progression of subclinical cardiac disease. Thus, our aim was to compare the ability of native T1 and extracellular volume (ECV) measurements to differentiate risk of myocardial disease in DMD and controls.

**Methods:**

Twenty boys with DMD and 16 age/gender-matched controls without history predisposing to cardiac fibrosis, but with a clinical indication for cardiovascular magnetic resonance (CMR) evaluation, underwent CMR with contrast. Data points collected include left ventricular ejection fraction (LVEF), left ventricular mass, and presence of late gadolinium enhancement (LGE). Native T1, and ECV regional mapping were obtained using both a modified Look-Locker (MOLLI) and saturation recovery single shot sequence (SASHA) on a 1.5T scanner. Using ordinal logistic regression models, controlling for age and LVEF, LGE-free septal we evaluated the ability native T1 and ECV assessments to differentiate levels of cardiomyopathy.

**Results:**

Twenty DMD subjects aged 14.4 ± 4 years had an LVEF of 56.3 ± 7.4 %; 12/20 had LGE, all confined to the lateral wall. Sixteen controls aged 16.1 ± 2.2 years had an LVEF 60.4 ± 5.1 % and no LGE. Native T1 and ECV values were significantly higher in the DMD group (*p* < 0.05) with both MOLLI and SASHA imaging techniques. Native T1 demonstrated a 50 % increase in the ability to predict disease state (control, DMD without fibrosis, DMD with fibrosis). ECV demonstrated only the ability to predict presence of LGE, but could not distinguish between controls and DMD without fibrosis.

**Conclusions:**

LGE-spared regions of boys with DMD have significantly different native T1 and ECV values compared to controls. Native T1 measurements can identify early changes in DMD patients without the presence of LGE and help predict disease severity more effectively than ECV. Native T1 may be a novel outcome measure for early cardiac therapies in DMD and other cardiomyopathies.

## Background

Duchenne muscular dystrophy (DMD) is an X-linked disorder affecting 1:3500 to 1:6000 male births worldwide, causing severe disability and death due to cardiopulmonary failure associated with dilated cardiomyopathy and restrictive pulmonary disease [1]. The dilated cardiomyopathy onset and progression are variable; however severe congestive heart failure is nearly universal by adulthood [2, 3]. Current efforts to improve both lifespan and quality of life in this disease are targeted towards delaying the onset of cardiac remodeling and subsequent dysfunction [4].

The design and evaluation of early prophylactic therapies to prevent DMD cardiomyopathy require quantitative measures of left ventricular (LV) remodeling to detect early, subclinical myocardial changes and monitor effectiveness [5]. However, current measures of LV remodeling, such as ECG [6], echocardiography evaluation including diastolic indices and myocardial strain imaging [7], and serum biomarkers [8], are limited. Cardiovascular magnetic resonance (CMR) offers additional diagnostic benefits in the assessment of myocardial changes present in cardiomyopathy. CMR using late gadolinium enhancement (LGE) imaging is able to show areas of edema and fibrosis [9, 10], however, the extent of disease can be underestimated in conditions where the myocardium is affected globally, such as DMD cardiomyopathy [11].

T1-mapping by CMR is an emerging technique, which offers the ability to quantify myocardial fibrosis. Widely used protocols for T1-mapping in the heart are based on either inversion or saturation recovery using imaging sequences such as Modified Look-Locker (MOLLI) and Saturation recovery single shot acquisition (SASHA), respectively. Baseline normal values of T1 are different for different methods [12], and there is current debate on the pros and cons for each technique [13]. Pre- and post-contrast T1 can be measured and combined for the assessment of extracellular volume (ECV) for detection and quantification of diffuse myocardial fibrosis [12, 14, 15]. However, with new concerns regarding gadolinium accumulation in the brain [16], pre-contrast, or”native” T1 measurements have been studied and shown to provide useful clinical data without contrast administration [17, 18].

The ability to detect and follow subclinical myocardial fibrosis using noninvasive imaging may be a powerful tool to monitor response to early prophylactic myocardial therapies. Therefore, our aim was to compare native T1 and extracellular volume (ECV) measurements in DMD and controls, and evaluate their ability to stratify myocardial disease.

## Methods

With IRB approval and informed consent/assent, 20 boys with DMD who were not mechanically ventilated underwent CMR with gadolinium contrast on a Siemens Aera 1.5T MR scanner (Siemens Healthcare, Erlangen, Germany). The CMR study included volumetric analysis to obtain left ventricular ejection fraction (LVEF) and fat-water separation imaging to exclude significant intra-cardiac deposits of fat which would alter T1 values [19]. Native T1 mapping was performed in four short axis slices (excluding apical regions to avoid partial volume effect) and a four chamber slice using both a MOLLI and SASHA sequence. Following intravenous administration of gadobutrol 0.15 mmol/kg, late gadolinium enhancement (LGE) imaging and post-contrast T1 mapping were performed, again using both the MOLLI and SASHA methods in identical slice positions. The order of MOLLI and SASHA acquisition following contrast administration was randomized and all post-contrast T1 maps were acquired between 15 and 22 min after contrast administration to assure that a dynamic equilibrium was achieved.

Additionally, and with IRB approval and informed consent/assent, 16 age-matched boys undergoing CMR with gadolinium for a separate clinical indication with normal results and no evidence of LGE were included in the analysis as a control group. These subjects underwent identical study procedures in terms of T1 mapping technique and gadolinium administration. Subjects with any history of cyanotic congenital heart disease, confirmed cardiomyopathy, history of cardiopulmonary bypass were excluded prior to imaging procedures, and subjects with calculated abnormal ejection fraction or evidence of late gadolinium enhancement were excluded from the control group following the imaging procedure.

### Parametric mapping

Maps of T1 values were generated using both the MOLLI and SASHA techniques for each of the 4 short axis slices and one four chamber slice. Table 1 lists sequence parameters for SASHA and MOLLI for heart rates less than 90 bpm and heart rates greater than 90 bpm. The MOLLI acquisition sampled the inversion recovery using a 5 s(3 s)3 s scheme for native T1 contrast and a 4 s(1 s)3 s(1 s)2 s scheme following contrast [13]. The SASHA sampling used a NS+[1(0)]^12^ sampling strategy at fixed delays of 600 ms pre-contrast and 200 ms post-contrast [20]. T1 maps were acquired with breath-holds due to the very precise nature of creating a T1 measurement. There is a motion-correction algorithm that aligns the various T1-weighted images of both the Look-Locker and the Saturation recovery sequences prior to map creation that works most optimally when through-plane motion associated with respiration is minimized, leaving the algorithm to adjust for any cardiac motion.

**Table 1:** Sequence parameters for T1 mapping using MOLLI and SASHA techniques

Sequence parameter	MOLLI	MOLLI	SASHA	SASHA
	HR < 90	HR > 90	HR < 90	HR > 90
FOV (mm)	360 × 270	360 × 270	360 × 270	360 × 270
Matrix	256 × 144	192 × 120	256 × 144	192 × 120
Resolution (mm)	1.4 × 1.9	1.9 × 2.3	1.4 × 1.9	1.9 × 2.3
Slice thickness (mm)	8	8	8	8
TE (msec)	1.12	1.01	1.12	1.01
TR (msec)	2.7	2.44	2.7	2.44
Flip angle (^o^)	35	35	70	70
Acquisition window (msec)	167	126	167	126
Parallel imaging acceleration	2	2	2	2
Partial Fourier	7/8	7/8	7/8	7/8

Sequence parameters for T1 mapping using MOLLI and SASHA techniques, which were tailored to the subjects’ baseline heart rate in order to optimize resolution for HR < 90, and HR > 90 for each technique. *FOV* field of view, *TE* echo time, *TR* repetition time

Following the CMR exam, maps of ECV values were created for each of the five slice positions (four short axis slices and 1 four chamber slice) on a per-pixel basis using the pre-contrast T1 map, the post-contrast T1 map, and the venous hematocrit at the time of IV placement for the CMR [15].

### T1 and ECV measurements

**Fig. 1:**
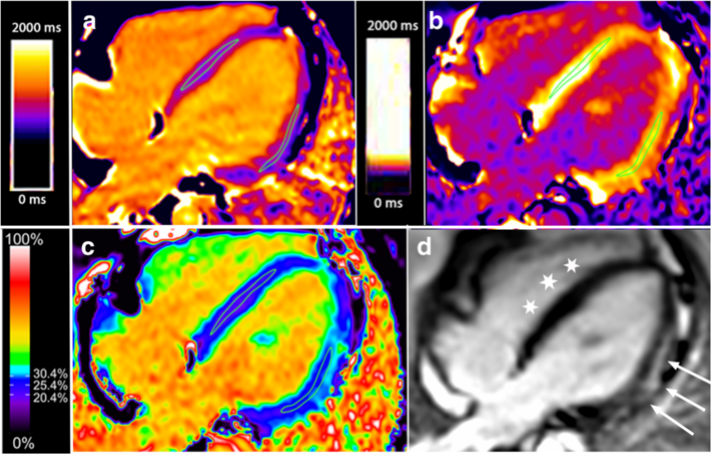
Four chamber view of a boy with DMD and lateral wall fibrosis using MOLLI. Panel **a** is the native T1 map, panel **b** is the post-contrast T1 map, panel **c** is the extracellular volume (ECV) map and panel **d** is the late gadolinium enhancement (LGE) map. Typical regions of interest to derive T1 and ECV values are displayed on the three parametric maps. *Arrows* indicate the lateral wall bright, subepicardial LGE, while stars indicate the nulled, normal septal myocardium

**Fig. 2:**
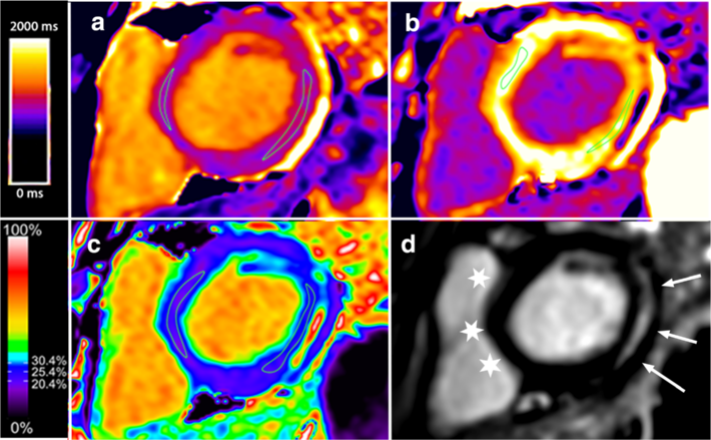
Short axis view of the same boy with DMD depicted in Fig. 1 with lateral wall fibrosis using MOLLI. Panel **a** is the native T1 map, panel **b** is the post-contrast T1 map, panel **c** is the extracellular volume (ECV) map and panel **d** is the late gadolinium enhancement (LGE) map. Typical regions of interest to derive T1 and ECV values are displayed on the three parametric maps. *Arrows* indicate the lateral wall bright, subepicardial LGE, while stars indicate the nulled, normal septal myocardium

Two regions of interest (ROI), one septal and one in the lateral wall, were generated for each of the five slice positions using the “middle-third” technique to generate an average, regional pixel value from that parametric map. Care was taken to avoid including pixels containing blood pool at the endocardial border, epicardial fat, and any artifacts in the ROI. Regions of late gadolinium enhancement were generally avoided due to their typical subepicardial location in the lateral wall when visible, which is not included in the standard “middle third” technique of ROI generation. This benefits the analysis in that it is easier to determine the added value of parametric mapping beyond identification of LGE. However, patchy widespread LGE cannot be excluded, and so was included when present. Figure 1 demonstrates a typical four chamber slice position, with native T1 map, post-contrast T1 map and ECV map with ROI included, and Fig. 2 demonstrates a short axis slice from the same patient. Mean and SD of the native T1, post-contrast T1 and ECV were noted for the septal and lateral positions for each SAX and 4CH slice.

### Analysis

Mean values of age, LV mass, LVEF, septal and lateral native T1, post contrast T1 and ECV from the DMD and control subjects were tallied and compared using a paired, 2-tailed t-test. Presence and location of intramyocardial fat and LGE were noted.

Ordinal logistic regression models were developed to evaluate the ability of native T1 and ECV to distinguish among the three clinical groups: normal controls, DMD free of fibrosis, and DMD with fibrosis. Controlling for small group differences in age and LVEF, these models were used to derive group membership profiles based on estimates with 95 % confidence intervals of the probability of membership in each group with increasing levels of each imaging procedure. All statistical analyses were completed with Stata 13 (StataCorp, College Station, TX).

## Results

The DMD subjects’ (*n* = 20) average age was 14.4 ± 4 years, average LVEF was 56.3 % ± 7.4 % (range 39–66 %) and 12/20 had evidence of LGE. All LGE was patchy, subepicardial and was confined to the lateral wall of the left ventricle; thus all septal myocardium in the DMD cohort appeared normal (nulled) on LGE imaging. No regions of intramyocardial fat deposition were noted on fat-water separation imaging. The male controls (*n* = 16) had an average age of 16.1 ± 2.2 years with an LVEF 60.4 ± 5.1 % and no LGE. Study indications for the controls were to rule out arrhythmogenic cardiomyopathy, evaluate the thoracic aorta, and evaluate the right ventricle post pulmonary balloon valvuloplasty (with a normal RV mass, RV volumes and RV function and mild pulmonary regurgitation). None had undergone cardiopulmonary bypass, were cyanotic or have confirmed or suspected myocarditis. Table 2 displays demographic information for the two groups. A summary of the degree of cardiopulmonary disease is listed in Table 3, including current glucocorticoid/cardiac therapies and most recent pulmonary function testing for the DMD cohort.

**Table 2:** Demographic table of DMD and control subjects, with further breakdown of the DMD group into DMDLGE+ and DMDLGE -

	Control	DMD	*p*	DMD	DMD	*p*
				LGE –	LGE +	
	*n* = 16	*n* = 20		*n* = 8	*n* = 12	
Age (years)	16.1 ± 2.2	14.4 ± 4	0.15	12.3 ± 3.7	15.8 ± 4.1	
LVEF (%)	60.4 ± 5.1	56.3 ± 7.4	0.07	61.9 ± 3.6	52.6 ± 6.9	
LVMi (g/m^2^)	70.2 ± 18.0	53.1 ± 17.6	0.01	47.5 ± 10	56.8 ± 20.2	
LVEDVi (ml/m^2^)	91 ± 36	105 ± 16	0.10	78.4 ± 34.5	98.7 ± 42.7	
% LGE	0	60 %	--	0	100	--
% HR > 90 bpm	24 %	65 %	--			--

*LVEF* left ventricular ejection fraction, *LVMi* left ventricular mass index, *LVEDVi* left ventricular end-diastolic volume indexed

**Table 3:** Glucocorticoid and cardiac medications and respiratory data for DMD subjects at time of CMR

DMD Subject	Glucocorticoid/ Cardiac Medications	FVC%	FEV1%	Nighttime support	Restrictive lung disease
1	Deflazacort	76 %	83 %	None	None
2	Prednisone, Lisinopril	54 %	44 %	None	Mild to moderate
3	Lisinopril	57 %	58 %	BiPAP	Mild
4	Lisinopril	55 %	65 %	None	Mild
5	Prednisone	116 %	115 %	None	None
6	Prednisone	27 %	32 %	BiPAP	Severe
7	Deflazacort	61 %	67 %	BiPAP	Moderate
8	Prednisone, Perindopril	55 %	59 %	None	Moderate
9	Prednisone, Lisinopril	57 %	54 %	None	Mild to moderate
10	Prednisolone	72 %	71 %	None	Mild
11	None	79 %	61 %	None	Mild
12	Prednisone, Perindopril	60 %	67 %	None	Mild to moderate
13	Deflazacort	121 %	111 %	None	None
14	Prednisone	92 %	92 %	None	None
15	Prednisolone	102 %	106 %	None	None
16	Prednisone, Lisinopril	58 %	67 %	BiPAP	Mild to moderate
17	Prednisolone	94 %	105 %	None	None
18	Prednisone, Perindopril	74 %	86 %	BiPAP	Mild
19	Deflazacort, Lisinopril	104 %	93 %	None	None
20	Prednisone, Lisinopril	46 %	50 %	BiPAP	Moderate to severe

*DMD* Duchenne muscular dystrophy, *FVC* functional vital capacity, *FEV* forced exhaled volume

### Effect of T1 mapping technique

**Fig. 3:**
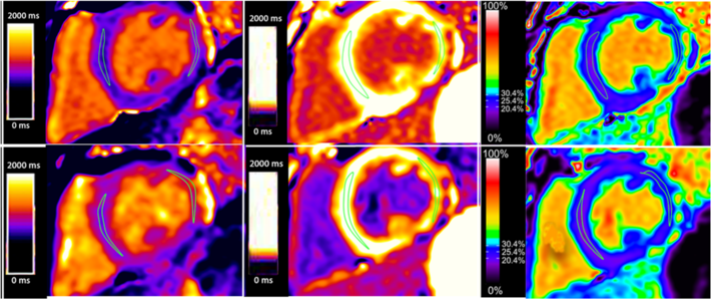
Comparison of an identical short axis slice using MOLLI (*top row*) and SASHA (*bottom row*). Pixel color represents the T1 value in the native T1 maps (*left*) and post-contrast T1 maps (*middle*), and pixel color represents the ECV value in the ECV map (right). There is a slight trend towards higher T1 and lower ECV values in SASHA as compared to MOLLI

Table 4 contains average +/- SD values for native T1, post-contrast T1 and ECV values for each population by MOLLI and SASHA. Differences are identified in native T1, post-contrast T1 and ECV between DMD and controls (all *p* < 0.05, Table 3). These differences are statistically significant regardless of T1 mapping strategy (MOLLI or SASHA), and regardless of the presence of LGE within the ROI, as both the lateral wall (contains all LGE) and septal (does not contain LGE). Figure 3 displays both the MOLLI and SASHA-derived parametric maps for a single slice. Absolute values of T1 were roughly 120 % of the MOLLI values, and absolute values of ECV were roughly 80 % of the MOLLI.

**Table 4:** Native T1 and ECV values for subjects with DMD and normal controls

Technique	Location	Measurement	DMD^a^	DMD^a^	Control	Control	*p*
			Average	SD	Average	SD	
MOLLI	Septal	Native T1	1046.1	61.1	989.9	33.5	0.000
	Septal	ECV^b^	27.9	5.8	26.0	3.3	0.047
	Lateral	Native T1	1075.1	71.8	978.2	36.4	0.000
	Lateral	ECV^b^	31.3	6.7	24.4	3.5	0.000
SASHA	Septal	Native T1	1233.6	66.8	1133.6	72.0	0.000
	Septal	ECV^b^	23.6	4.3	20.0	2.6	0.008
	Lateral	Native T1	1268.6	78.2	1135.8	144.2	0.001
	Lateral	ECV^b^	27.5	6.5	21.4	2.3	0.000

Native T1 and ECV values for subjects with DMD and normal controls in the septal and lateral locations, using both the MOLLI and SASHA techniques for measuring T1. Septal myocardial values of ECV are significantly different between DMD and control subjects, using both imaging methods. In this cohort of boys with DMD, all LGE was contained to the septum, thus this finding represents a significant difference in myocardium that does not have overt fibrosis on LGE imaging.^a^DMD Duchenne muscular dystrophy, ^b^ECV extracellular volume fraction

### Effect of age, LVEF and LGE within the DMD cohort

Table 5 compares septal measurements of native T1 and ECV in the DMD cohort by age (>14 vs. ≤ 14 years), LVEF (>55 % vs ≤ 55 %) and presence of fibrosis (LGE+ vs LGE-) based on t-tests. There are few statistically significant differences between native T1 and ECV values in these groups. Two exceptions are noted, including differences by LVEF (>55 % vs. ≤55 %) in the septal ECV level in MOLLI and SASHA (*p* = 0.06, *p* = 0.02, respectively), and differences by age (>14 vs. ≤14 years) in septal native T1 using MOLLI. The native T1 values are higher in the younger group, counter to what would be otherwise expected, which may be a result of the small sample size. The lateral wall analysis does not show significant differences between the older and younger cohorts, and there are significant differences between the LGE + and LGE – groups in nearly all measurements. Table 6 contains identical information for the lateral wall, where all LGE was contained if present. Identified are expected, statistically significant differences in lateral ECV and lateral native T1.

**Table 5:** Septal values of native T1 and ECV within subgroups of the DMD cohort

**Technique**	**Location**	**Measurement**	**Age < 14**		**Age > 14**		
			**Average**	**SD**	**Average**	**SD**	***p***
MOLLI	Septal	Native T1	1054.69	32.04	1040.40	73.98	0.20
	Septal	ECV^a^	27.57	4.40	28.04	6.49	0.67
SASHA	Septal	Native T1	1257.36	43.68	1217.37	74.60	<0.01
	Septal	ECV^a^	23.74	2.43	23.44	5.23	0.71
			**LVEF** ^**b**^ ** > 55 %**		**LVEF** ^**b**^ ** < 55 %**		
			**Average**	**SD**	**Average**	**SD**	**p**
MOLLI	Septal	Native T1	1040.32	37.79	1055.49	85.86	0.32
	Septal	ECV^a^	26.81	4.13	29.40	7.39	0.06
SASHA	Septal	Native T1	1230.34	58.48	1238.84	78.02	0.58
	Septal	ECV^a^	22.64	2.78	24.99	5.70	0.02
			**LGE** ^**c**^ **-**		**LGE** ^**c**^ **+**		
			**Average**	**SD**	**Average**	**SD**	**p**
MOLLI	Septal	Native T1	1039.49	39.62	1050.62	71.69	0.33
	Septal	ECV^a^	27.09	3.84	28.36	6.68	0.24
SASHA	Septal	Native T1	1223.85	55.55	1240.30	72.79	0.22
	Septal	ECV^a^	23.06	2.93	23.89	5.03	0.31

Septal values of native T1 and ECV using the MOLLI and SASHA techniques for measuring T1 within subgroups of the DMD cohort divided into those < 14 years (*n* = 12), and those > 14 years (*n* = 8); divided again into those with LVEF > 55 % (*n* = 12) and LVEF < 55 % (*n* = 8); divided again into those with LGE (*n* = 8) and those without LGE (*n* = 12). There are few significant differences between the group, including only septal ECV using MOLLI and SASHA when the group is divided by LVEF, and native T1 when the group is divided by age.^a^ ECV extracellular volume fraction, ^b^LVEF left ventricular ejection fraction, ^c^LGE late gadolinium enhancement

**Table 6:** Lateral values of native T1 and ECV within subgroups of the DMD cohort

**Technique**	**Location**	**Measurement**	**Age < 14**		**Age > 14**		
			**Average**	**SD**	**Average**	**SD**	***p***
MOLLI	Lateral	Native T1	1070.3	52.7	1078.4	82.3	0.56
		ECV^a^	29.6	5.5	32.4	7.1	0.03
SASHA	Lateral	Native T1	1279.7	76	1260.8	78.7	0.25
		ECV^a^	25.6	4.1	28.9	7.4	0.01
			**LVEF** ^**b**^ ** > 55 %**		**LVEF** ^**b**^ ** < 55 %**		
			**Average**	**SD**	**Average**	**SD**	***p***
MOLLI	Lateral	Native T1	1061.4	52.2	1096.6	90.8	0.04
		ECV^a^	29.3	5.0	34.4	7.7	<0.01
SASHA	Lateral	Native T1	1263.8	72.2	1276.4	86.5	0.47
		ECV^a^	24.9	4.1	31.7	7.3	<0.01
			**LGE** ^**c**^ **-**		**LGE** ^**c**^ **+**		
			**Average**	**SD**	**Average**	**SD**	***p***
MOLLI	Lateral	Native T1	1046.5	48.0	1094.8	78.6	<0.01
		ECV^a^	27.7	3.9	33.7	7.1	<0.01
SASHA	Lateral	Native T1	1255.4	75.8	1277.8	78.5	0.16
		ECV^a^	23.5	3.4	30.3	6.6	<0.01

Lateral values of native T1 and ECV using the MOLLI and SASHA techniques for measuring T1 within subgroups of the DMD cohort divided into those < 14 years (*n* = 12), and those > 14 years (*n* = 8); divided again into those with LVEF > 55 % (*n* = 12) and LVEF < 55 % (*n* = 8); divided again into those with LGE (*n* = 8) and those without LGE (*n* = 12). In this case, using regions of interest that would contain LGE if present, significant differences are found in ECV measurements across the board, and in Native T1 measurements using MOLLI. ^a^ ECV extracellular volume fraction, ^b^LVEF left ventricular ejection fraction, ^c^LGE late gadolinium enhancement

**Fig. 4:**
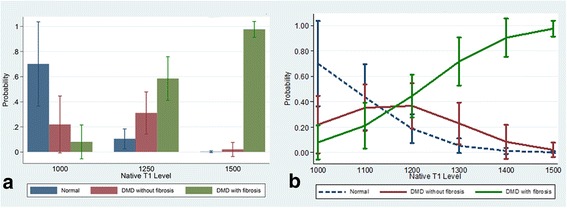
Probability (Y-axis) for a given value of LGE-free, native T1 (X-axis) as measured by the SASHA technique, of belonging to one of three groups, (1) normal, (2) DMD without LGE and (3) DMD with LGE. Panel **a** demonstrates the probabilities of each of the 3 groups bar graph form, grouped by T1 value range. Panel **b** shows a line graph for each of the 3 groups with 95 % CI bars of the probability as the T1 value increases. As the native T1 value increases from 1000 to 1500 the probability of being in the DMD with LGE group increases significantly, and the probability of being in the normal group goes to 0 with excellent discrimination between groups at T1 values less than 1000 and greater than 1110

**Fig. 5:**
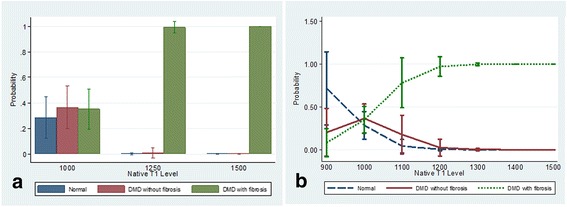
Probability (Y-axis) for a given value of LGE-free, native T1 (X-axis) as measured by the MOLLI technique, of belonging to one of three groups, (1) normal, (2) DMD without LGE and (3) DMD with LGE. Panel **a** demonstrates the probabilities of each of the 3 groups bar graph form, grouped by T1 value range. Panel **b** shows a line graph for each of the 3 groups with 95 % CI bars of the probability as the T1 value increases. As the native T1 value increases from 900 to 1500 the probability of being in the DMD group with the most advanced disease increases significantly, and the probability of being in the normal group goes to 0, with outstanding discrimination between groups at T1 values less than 900 and greater than 1250

**Fig. 6:**
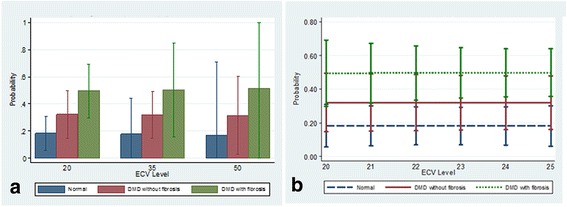
Probability (Y-axis) for a given value of LGE-free, ECV (X-axis) as measured by the SASHA technique, of belonging to one of three groups, (1) normal, (2) DMD without LGE and (3) DMD with LGE. Panel **a** demonstrates the probabilities of each of the 3 groups bar graph form, grouped by ECV value range. Panel **b** shows a line graph for each of the 3 groups with 95 % CI bars of the probability as the ECV value increases. There is no ability to discriminate the probability of belonging to one of the 3 groups based on ECV value derived from SASHA measurements

**Fig. 7:**
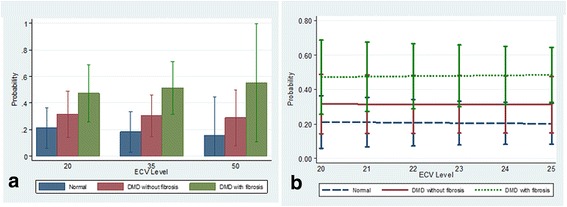
Probability (Y-axis) for a given value of LGE-free, ECV (X-axis) as measured by the MOLLI technique, of belonging to one of three groups, (1) normal, (2) DMD without LGE and (3) DMD with LGE. Panel **a** demonstrates the probabilities of each of the 3 groups bar graph form, grouped by ECV value range. Panel **b** shows a line graph for each of the 3 groups with 95 % CI bars of the probability as the ECV value increases. There is no ability to discriminate the probability of belonging to one of the 3 groups based on ECV value derived from MOLLI measurements

Figures 4, 5, 6 and 7 present clinical disease state probability profiles based on septal (LGE-free) views using native T1 and ECV levels derived from SASHA and MOLLI procedures after controlling for group differences in age and LVEF levels. Only the native T1 based on the SASHA procedure provides consistent, statistically significant evidence of groupwise differences. At the lower native T1 level (1000), only the normal controls are highly prevalent with a probability of ~70 % compared to the DMD groups with a probability of 21 % and <10 %, for those without and with fibrosis, respectively. At the higher native T1 level (1500), the DMD group with fibrosis predominates with a probability >95 %. In the native T1 midrange (1250), evidence of group discrimination still persists, with a probability of DMD with fibrosis of ~60 %, a probability of DMD without fibrosis of ~30 % and a probability of normality of ~10 %.

## Discussion

This study used CMR to demonstrate significant differences in the native T1and ECV measurements in the myocardium of DMD subjects compared to age-matched controls. Most importantly, this is the first study to demonstrate that native T1 measurements in LGE-negative myocardium are proportional to the degree of cardiovascular disease in the DMD cohort using regression analyses. Thus, native T1 values can be measured with CMR and used to assess myocardial changes, including fibrosis and inflammation, and disease state among myocardial segments that all appear “normal on LGE”, making this a viable, quantitative measure of subclinical disease without the use of contrast that could be used to monitor early cardiac therapies.

Initial studies focused on the presence of LGE in DMD patients using contrast-enhanced imaging techniques [21–23]. More recently, clinical implications of LGE in DMD were demonstrated when Florian et al. [24] showed that “transmural” LGE was associated with more adverse events in muscular dystrophy subjects and Tandon et al. [25] found correlations between the presence of LGE, LVEF and genotype. Soslow et al. also looked at post-contrast T1 ratios and found lower ratios in DMD subjects compared to controls, even in LGE negative subjects [26].

ECV is the most recent technique used to measure myocardial extracellular matrix expansion. Soslow et al. recently showed significantly increased global ECV values in DMD compared to controls, but no difference in global ECV in DMD subjects with and without LGE [27]. This current study confirms significantly elevated ECV values in DMD vs. controls, and in addition, LGE-free myocardium has a significantly higher ECV in DMD than in normals. While ECV can discriminate between DMD and controls, it performs poorly in the ability to distinguish disease severity within the DMD cohort. ECV demonstrated only the ability to predict presence of LGE, but couldn’t distinguish between controls and DMD without fibrosis.

With the recent concerns regarding frequent use of gadolinium contrast [16], a marker of subclinical myocardial disease without contrast is optimal. As with Soslow et al. (26), we demonstrated increased native T1 measures in DMD subjects compared to controls. Due to variability in T1 mapping sequences, this study also included SASHA sequences to further validate T1 mapping. We also studied septal native T1 measures within the DMD cohort based on age, LVEF and presence of LGE, but the average native T1 values did not differ significantly within these groups (Table 3). However, when we controlled for age and LVEF, native T1 measures using the SASHA protocol demonstrated an improved ability to stratify DMD subjects by disease severity (normal, DMD without fibrosis and DMD with fibrosis; Fig. 4). Native T1 determined by MOLLI demonstrated ability to only stratify the presence of fibrosis. Therefore, this study suggests the utility of native T1 measures, and not ECV, to stratify disease severity in DMD subjects without the use of contrast in a small cohort of subjects.

This study incorporated both MOLLI and SASHA imaging strategies to increase confidence of any observed differences in T1 values between the DMD and control groups, as such differences, if present, were expected to be subtle. This is based on a recent study that showed how measurement precision and accuracy can be affected by the T1 mapping technique chosen and MOLLI and SASHA were among the strongest techniques [28]. In terms of accuracy, SASHA values are known to be approximately 10–15 % higher than MOLLI and our data is consistent with this observation. Also, SASHA is known to be a less precise technique [13] and our SASHA measures have larger standard deviations compared to MOLLI. However, in the current study, SASHA showed a better ability in distinguishing clinical disease states. This may be because MOLLI is more susceptible to measurement imperfections due to high and/or variable heart rates which may have diminished the precision in a pediatric cohort. Based on these results, further studies are needed to determine if SASHA is a better technique to measure early, diffuse myocardial changes in DMD.

Limitations of this work include general limitations of T1 mapping techniques, which can yield variable results at higher heart rates, and are prone to partial-volume errors with hearts that are thin and small. Although T1 mapping sequences were generally tailored to the heart rate, heart rates above 100 bpm may affect T1 values. Additionally, although no overt intra-cardiac fat was noted on fat-water separation imaging; theoretically, microscopic fat deposition may be part of the DMD cardiomyopathy, which would affect the ECV and T1 mapping values in addition to cardiac fibrosis [29]. While prior studies have linked elevated T1 values with cardiac fibrosis, DMD myocardial disease may include an inflammatory component which may be altering T1 values in addition to fibrosis. Since no endomyocardial biopsies were obtained in this study, it is unclear how much of a role fibrosis vs. inflammation played in the T1 values of this study population. Another limitation is that the extent of LGE in the study population is more difficult to capture by T1 mapping compared to the severity of LGE, as the validity of T1 relies on exclusion of any questionable pixels from the ROI, thus extent will always be underestimated. The control population is a population of convenience, as acquiring contrast-enhanced imaging from known normal children is not practical or ethical due to need for IV placement and exposure to gadolinium without a clinical indication. Another limitation is the small sample size, which is typical for rare diseases [25] and can limit conclusions drawn from these results as the study was not powered to detect small differences amongst this heterogeneous disease. Finally, all T1 mapping was carried out on a single magnet, from a single vendor, and therefore has limited utility to the overall community of cardiologists involved in the care of all patients with DMD. Ideally, future work would focus on creating reference values for normal children at different magnet strengths and vendors, which would require multi-center pooling of data.

## Conclusions

This study is the first to demonstrate the novel utility of native T1 measures to stratify DMD patients by extent of myocardial disease. ECV measures, while increased in DMD compared to control, could not distinguish disease severity within the DMD cohort. Native T1 imaging provides a novel imaging biomarker for monitoring myocardial changes related to early subclinical disease in DMD and possibly other cardiomyopathies.

## Abbreviations

CMR: Cardiovascular magnetic resonance
DMD: Duchenne muscular dystrophy
ECV: Extracellular volume
LGE: Late gadolinium enhancement
LVEF: Left ventricular ejection fraction
MOLLI: Modified look locker
ROI: Region of interest
SASHA: Saturation recovery single shot acquisition
